# Risk Factors for Hemorrhagic and Ischemic Stroke in Sub-Saharan Africa

**DOI:** 10.1155/2018/4650851

**Published:** 2018-05-31

**Authors:** Gertrude Namale, Onesmus Kamacooko, Alison Kinengyere, Laetitia Yperzeele, Patrick Cras, Edward Ddumba, Janet Seeley, Robert Newton

**Affiliations:** ^1^MRC/UVRI and LSHTM Uganda Research Unit, Entebbe, Uganda; ^2^Africa Centre for Systematic Reviews and Knowledge Translation, College of Health Sciences, Makerere University, Kampala, Uganda; ^3^University of Antwerp, Department of Neurology, Antwerp, Belgium; ^4^St. Francis Hospital Nsambya Affiliated to Uganda Martyrs University, Kampala, Uganda; ^5^London School of Hygiene & Tropical Medicine, London, UK; ^6^University of York, UK

## Abstract

**Introduction:**

In sub-Saharan Africa (SSA), there is a significant burden of ischemic stroke (IS) and hemorrhagic stroke (HS), although data on risk factors for each type are sparse. In this systematic review we attempt to characterize the risk factors.

**Methods:**

We systematically reviewed (PubMed, EMBASE, WHOLIS, Google Scholar, Wiley online, and the Cochrane Central Register of Controlled Trials (CENTRAL)) case-control studies and case series from 1980 to 2016 that reported risk factors for IS and/or HS in SSA. For each risk factor we calculated random-effects pooled odds ratios (ORs) for case-control studies and pooled prevalence estimates for case series*. Results*. We identified 12 studies, including 4,387 stroke patients. Pooled analysis showed that patients who had diabetes (OR = 2.39; 95% CI: 1.14–5.03) and HIV (OR = 2.46 (95% CI: 1.59–3.81) were at a significantly greater risk of suffering from all stroke types. There were insufficient data to examine these factors by stroke type. Among case series, the pooled prevalence of hypertension was higher for HS than for IS (73.5% versus 62.8%), while diabetes mellitus (DM) and atrial fibrillation (AF) were more prevalent among IS compared to HS (15.9% versus 10.6% and 9.6% versus 2.3%, respectively).

**Conclusions:**

There remain too few data from SSA to reliably estimate the effect of various factors on the risk of IS and HS. Furthermore, the vast majority of cases were identified in hospital and so are unlikely to be representative of the totality of stroke cases in the community.

## 1. Background

Worldwide, stroke is a leading cause of death and of chronic disability [1]. It is estimated that 15 million people suffer a stroke annually; of these, five million die and another five million are left permanently disabled, placing a burden on family and community [2]. Approximately 85% of deaths are in low and middle income countries [3]. In SSA stroke represents an important part of the chronic disease burden [4], but there are relatively few data on risk factors.

Stroke is a heterogeneous disease comprising subarachnoid hemorrhage, intraparenchymal hemorrhage, and IS, each of which has a very different pathophysiology [5]. HS is thought to account for 15–20% of strokes worldwide, while IS accounts for 80–85%, although there are geographic variations [6]. In SSA, several nonmodifiable risk factors for stroke, such as age, gender, race, ethnicity, and heredity have been identified [7–9]. Potentially modifiable risk factors include hypertension, AF, hyperlipidemia, DM, cigarette smoking, physical inactivity, and transient ischemic attack (TIA) [7, 8]. Although the risk factors for HS and IS appear to vary considerably between countries [10], hypertension remains the most important stroke risk factor globally [11]. The INTERSTROKE study demonstrated the commonality of the main risk factors for stroke in SSA: hypertension (37%), alcohol intake (11%), physical inactivity (12%), and DM (12%) [12]. For example in Uganda, Ethiopia, and Zambia, some cross-sectional studies have shown a prevalence of stroke risk factors typical of countries in sub-Saharan Africa [13–17]. Similar trends were found in Sierra Leone, Tanzania, Kenya, Zimbabwe, Ethiopia, and Nigeria where hypertension was established to be the most common stroke risk factor, followed by diabetes mellitus, smoking, and alcohol intake [8, 12, 18–22].

However, there are relatively few data from SSA that examine risk factors for stroke and in particular for HS and IS separately. Here, we systematically review the available data, thereby summarizing what is known and highlighting gaps in knowledge on a continent that is undergoing rapid epidemiological transition.

## 2. Methods

### 2.1. Eligibility Criteria

Articles which were included in the review met the following inclusion criteria: (1) cohort, case-control, cross-sectional, or randomized control trial study design; (2) studies reporting risk factors for HS and IS with effect measures and 95% confidence intervals and used multivariable data analysis; (3) studies with data collected among adult patients aged 18 years and older; (4) studies conducted in SSA countries; (5) studies whose participants were clinically diagnosed with stroke as defined by the WHO criteria; (6) where stroke subtype was classified, it was by computer tomography (CT), magnetic resonance (MR), and brain imaging or necropsy; (7) articles published in English from 1980 to 2016; (8) studies from both community and hospital-based settings of first-ever as well as recurrent stroke.

**Figure 1:**
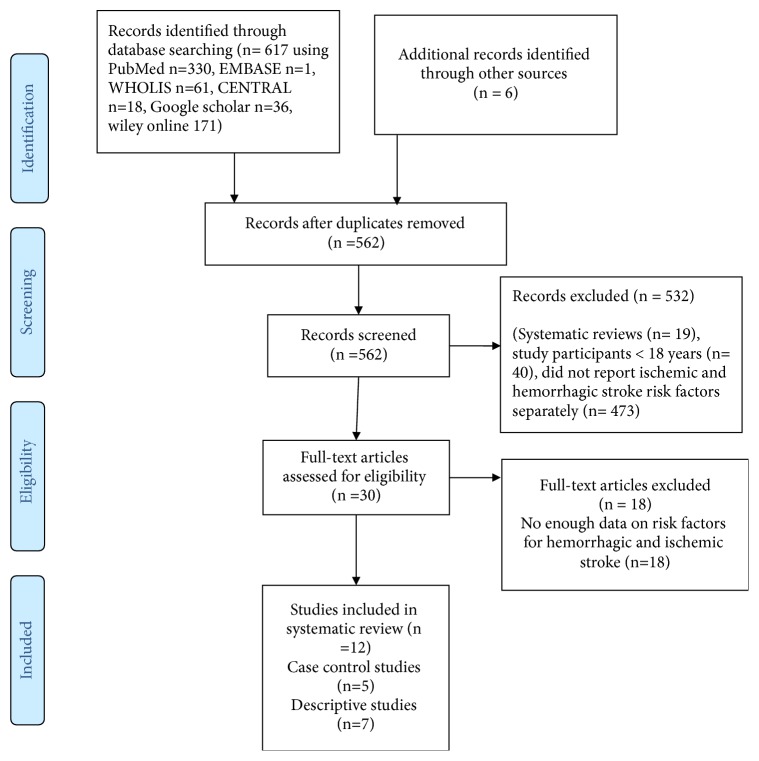
PRISMA flow diagram for the systematic review of risk factors for hemorrhagic and ischemic stroke in sub-Saharan Africa.* Footnotes*. The flow diagram template was adopted from the PRISMA statement [23].

The exclusion criteria were as follows: (1) the original article did not have relevant information on risk factors for HS and IS; (2) duplicate report; (3) results presented only as abstracts; (4) population not living in SSA; (5) studies including children; (6) studies with unclear or no available information on risk factors in all stroke or individual stroke types or data inconsistencies. Full text/papers were sought for all studies appearing to meet the inclusion criteria based on screening of the abstract, and a final selection was made by two independent reviewers.  Figure 1 shows a flow chart which was produced to facilitate transparency of the process.

### 2.2. Search Strategy

Standardised and well described methods were used in this systematic review [23]. Briefly, a search strategy was developed after identification of the relevant Medical Subject Headings (MESH), keywords, and their synonyms. The searches were conducted in six main databases: PubMed, EMBASE, WHOLIS, Google Scholar, Wiley online, and CENTRAL.

The search strategy for PubMed: the key words which we used in our search included terms describing stroke, terms describing age, terms describing the SSA countries, and terms describing risk factors as shown in the search strategy as follows:

1. (Stroke[Title] OR “Ischemic stroke”[Title] OR “Ischaemic stroke”[Title] OR “haemorrhagic stroke”[Title] OR “hemorrhagic stroke” OR “Cerebral Vascular accident” OR CVA)
2. (Adults OR “18 years or older”)
3. (“Sub-Saharan Africa”[Text Word] OR “Africa South of the Sahara” OR Angola[Text Word] OR Benin[Text Word] OR Botswana[Text Word] OR Burkina Faso[Text Word] OR Burundi[Text word] OR Cameroon[Text Word] OR “Cape Verde” OR “Central African Republic” OR Chad[Text Word] OR Comoros[Text Word] OR Congo (Democratic Republic) OR Congo (DRC- Kinshasa)[Text Word] OR “Côte d'Ivoire” OR Djibouti[Text Word] OR Eritrea[Text word] OR “Equatorial Guinea” OR “Congo Brazzaville” OR Ethiopia[Text Word] OR Gabon[Text word] OR Gambia[Text word] OR Ghana[Text Word] OR Guinea[Text Word] OR Guinea-Bissau[Text word] OR “Ivory Coast”[Text Word] OR Kenya[Text Word] OR Lesotho[Text Word] OR Liberia[Text word] OR Madagascar[Text Word] OR Malawi[Text Word] OR Mauritius[Text Word] OR Mozambique[Text Word] OR Namibia[Text Word] OR Niger[Text Word] OR Nigeria[Text Word] OR Réunion[Text word] OR Rwanda[Text Word] OR “Sao Tome and Principe”[Text word] OR Senegal[Text Word] OR Seychelles[Text word] OR “Sierra Leone”[Text Word] OR Somalia[Text word] OR “South Africa”[Text Word] OR Sudan[Text word] OR Swaziland[Text Word] OR Tanzania[Text Word] OR Togo[Text Word] OR Uganda[Text Word] OR Zambia[Text Word] OR Zimbabwe[text word])
4. (Hypertension OR “High blood pressure” [Text Word] OR Diabetes[Text Word] OR “Diabetes mellitus” OR Smoking OR Alcohol OR Obesity OR “Atrial fibrillation” [Text Word])
5. #1 AND #2 AND #3 AND #4 Limits: English language

The search yielded 617 abstracts. All abstracts were screened, and we assessed full papers of all abstracts meeting the inclusion criteria. We reviewed all relevant articles in English from 1980 to 2016. Stroke was defined as “rapidly developing clinical signs of focal, or at times, global disturbance of cerebral function, lasting more than 24 hours or leading to death with no apparent cause other than vascular origin” [2] as per the WHO criteria.

An additional search was conducted in reference lists of relevant studies, journals, theses, and conference proceedings to identify publications that could have been omitted in the database searches.

### 2.3. Study Selection

Two review authors (GN, AK) independently screened titles and abstracts of records obtained from the electronic searches and excluded those that were obviously irrelevant. We obtained the full text of the remaining studies and selected those for inclusion according to the above criteria. If any methodological question raised doubts about whether the study met the inclusion criteria, we contacted the study authors for clarification. If there was disagreement regarding the selection of studies, we attempted to reach a consensus. We recorded reasons for exclusion (Figure 1).

### 2.4. Data Extraction and Quality Assessment

Data were extracted from identified studies using a structured form by the primary reviewer (GN). A second reviewer (AK) independently checked the data extraction forms for accuracy and detail. We extracted the following information: (1) general information such as author, date of publication, and country; (2) study duration and sample size; (3) total number of participants, gender, and stroke type; (4) risk factors for stroke such as hypertension, DM, smoking, alcohol use, dyslipidaemia, HIV infection, and hypercholesterolemia. Two investigators (GN and OK) independently evaluated the methodological quality and risk of bias of eligible studies by using the Newcastle-Ottawa scale [28] and the Preferred Reporting Items for Systematic Reviews and Meta- Analyses (PRISMA) guidelines [23]. The studies were assessed on selection bias, definition of cases and controls, representativeness of the cases and controls, comparability of cases and controls, and ascertainment of exposure. Disagreements between two authors were resolved by discussions and if this was not possible, another senior investigator (RN) was consulted and participated in the discussions until consensus was reached.

### 2.5. Statistical Analyses

For each risk factor, where data were available from more than one study, we performed meta-analyses. We calculated study-specific odds ratios and pooled prevalence in HS and IS patients with 95% confidence intervals (CIs) separately. We used a random-effects model for meta-analyses since there was evidence of heterogeneity [29]. Heterogeneity among studies was assessed visually with a forest plot and quantitatively with the *I*^2^ index. The threshold of heterogeneity was set at *I*^2^ ≥ 50%. All meta-analyses were performed with STATA version 12.0 and REVMAN. Statistical significance was set at *p* ≤ 0.05. To assess whether pooled prevalence and ORs for each risk factor comparison differed between HS and IS, we assessed between-group heterogeneity, using the within-group pooled estimates and their standard errors.

## 3. Results

The literature search identified 617 publications from PubMed (330), EMBASE (1), WHOLIS (61), CENTRAL (18), Google scholar (36), and Wiley online (171). Additional 6 studies were included from reference lists of relevant publications. Five hundred and sixty-two (562) studies remained after removing 61 duplicates. After screening the titles and abstracts for relevance, 532 studies were excluded (19 were systematic reviews, 40 were among participants < 18 years, and 473 studies did not report data either on ischemic or hemorrhagic stroke risk factors separately) giving a total of 30 full texts that were assessed. After applying quality criteria, another 18 were excluded which did not have enough information on risk factors for hemorrhagic and ischemic stroke. A total of 12 studies were finally retained for the review. A summary of the selection process is shown in Figure 1.

### 3.1. Characteristics of the Case-Control Studies Included in the Meta-Analysis

There were five case-control studies [8, 12, 30–32] included in the final meta-analysis. Three [30–32] were single centre studies, while two [8, 12] were multicentre studies. All were matched case-control studies and reported information on more than one risk factor. The sample size ranged from 163 to 646. The total sample size from all retained studies was 2,384. Four studies used computed tomography (CT) scan for case ascertainment to confirm diagnosis while one study [30] used clinical assessment. Three studies reported information on the association between hypertension and stroke: four on diabetes and stroke, four on alcohol use and stroke, four on smoking and stroke, three on HIV infection and stroke, and two on hypercholesterolemia and stroke. Of the 5 studies, only one reported risk factors for HS and IS separately.  Table 1 summarizes the characteristics of the included studies.

### 3.2. Characteristics of Case Series Included in the Quantitative Synthesis

Seven studies [15–17, 24–27] were included in the final synthesis. All were conducted in hospital-based settings. The total sample size from all retained studies was 2003 with the mean of 286 (SD 195) and the median of 250 (IQR 139, 432). The pooled mean age for HS was 53.84 (SD 2.42) while the mean age for IS was 58.7 (SD 6.1). All seven studies used CT scan for case ascertainment to confirm diagnosis, two studies [26, 27] used both MRI and CT scan, while only one study [24] used both CT scan and necropsy. All seven studies reported risk factor data for HS and IS, but only one [17] also reported other risk factor data on TIA and other stroke types (Table 2).

### 3.3. Analysis of Case-Control Studies

**Figure 2:**
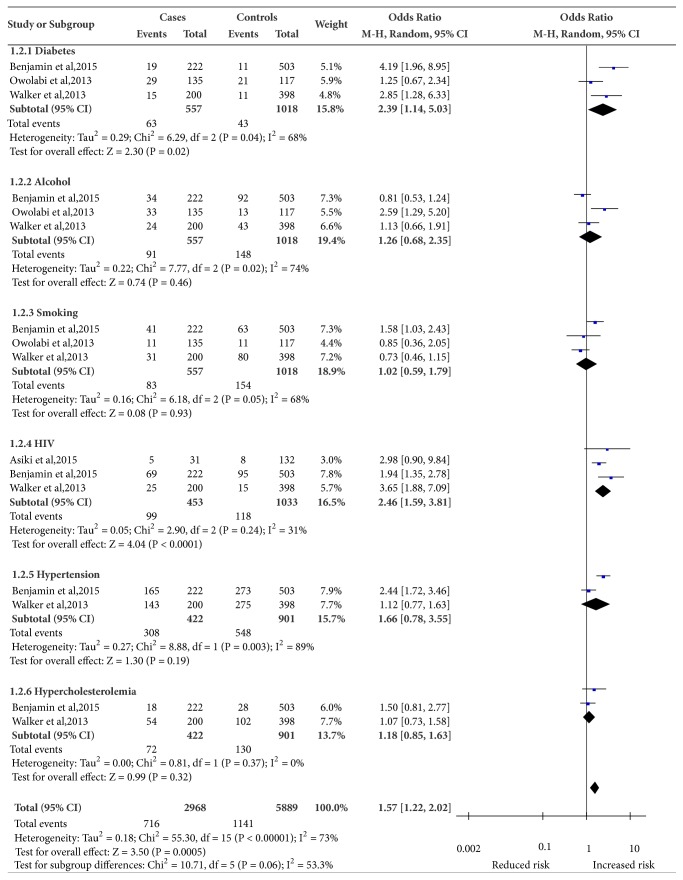
A meta-analysis of risk factors for all stroke in sub-Saharan Africa.

Among five case-control studies [8, 12, 30–32], we examined six risk factors for stroke: hypertension, DM, alcohol use, HIV infection, smoking, and hypercholesterolemia. These were risk factors with data available from more than one study. These five showed moderate to substantial heterogeneity between studies; hence random-effects models rather than fixed-effects were used to pool the ORs. A meta-analysis of risk factors for all stroke is summarized in Figure 2. Only one study [8] reported data on risk factors for HS and IS separately and so we could not perform a meta-analysis [33]. The risk factor associations with stroke are summarized in Table 3.

### 3.4. Diabetes Mellitus

Four studies [8, 12, 31, 32] reported relevant data on DM and its association with all stroke. Of these, only one study [8] assessed the associated risk of diabetes with HS and IS. Using a random-effects model, the pooled OR was 2.39 (95% CI: 1.14–5.03) for all strokes. There was substantial heterogeneity among studies (**I**^2^  =  68.0). One study [8] reported that DM was associated with an increased risk of IS (OR = 1.60; 99% CI: 1.29–1.99) compared to HS.

### 3.5. Alcohol Use

The effect of alcohol use on stroke risk was reported in four studies [8, 12, 31, 32]. Only one study [8] reported the risk on HS and IS. There was substantial heterogeneity among studies (**I**^2^  =  74.0%) in study effect size estimates. Using a random-effects model, the pooled OR was 1.26 (95% CI: 0.68–2.35). However one study [8] reported that history of alcohol intake of 1–30 drinks per month was associated with a reduced risk of IS (OR = 0.79; 99% CI: 0.63–1.00), while, for intracerebral HS, the risk increased with alcohol intake (OR 2.01; 99% CI: 1.35–2.99).

### 3.6. Smoking

Four studies [8, 12, 31, 32] reported relevant data on smoking and its effect on all stroke risk and again, only one [8] reported the risk with HS and IS. In the analysis both current and ever smoking were considered. There was considerable heterogeneity among studies (**I**^2^  =  68.0). Using a random-effects model the pooled OR was 1.02 (95% CI: 0.59–1.79). However one study [8] reported that smoking was associated with an greater risk of IS (OR 2.33, 99% CI, 1.91–2.81) compared to HS (OR 1.45, 99% CI, 1.07–1.96).

### 3.7. HIV Infection

Three studies [12, 30, 32] reported relevant data on HIV infection and its association with all stroke. There was moderate heterogeneity among studies (**I**^2^  =  31.0%). The pooled OR was 2.46 (95% CI: 1.59–3.81).

### 3.8. Hypertension

The risk of stroke with hypertension was reported in three case-control studies [8, 12, 32]; only one study [8] reported data on the risk with HS and IS. There was considerable heterogeneity among studies (*I*^2^ = 89.0%) in study effect size estimates and random-effects pooled OR was 1.66 (95% CI: 0.78–3.55). One study [8] reported that self-reported hypertension or high blood pressure > 160/90 mm Hg was the strongest risk factor for all stroke (OR = 3.80; 99% CI: 3.33–4.54), but more so for HS (OR = 9.18; 99% CI: 6.80–12.39) than for IS (OR = 3.14; 99% CI: 2.67–3.71).

### 3.9. Hypercholesterolemia

Two studies [12, 32] assessed the risk of hypercholesterolemia and all stroke. Using a fixed-effects model, the pooled OR was 1.18; 95% CI: 0.85–1.63. There was no heterogeneity among studies (**I**^2^ = * *0.0%).

### 3.10. Analysis of the Case Series

Because there were insufficient data from case-control studies to examine risk factors for hemorrhagic and ischemic stroke separately, we performed additional quantitative analyses using seven hospital-based descriptive studies [15–17, 24–27]. These studies were only reporting prevalences of risk factors for HS and IS cases. We examined seven risk factors for HS and IS which included hypertension, DM, AF, alcohol use, HIV infection, smoking, and hypercholesterolemia. These were risk factors with data available from more than one study. The overall pooled prevalence of IS was 61.4% while HS was 33%. The pooled prevalence of hypertension was higher for HS than for IS (73.5% versus 62.8%), while DM and AF were more prevalent among IS compared to HS (15.9% versus 10.6% and 9.6% versus 2.3%, respectively). The summary of the pooled prevalences for HS and IS by selected risk factors for stroke is summarized in Table 4.

## 4. Discussion

There remain too few data from SSA to reliably estimate the effect of various factors on the risk of IS and HS. In this review, we identified only five case-control studies from SSA that reported information on risk factors for all stroke. Only one study reported data on IS and HS risk factors separately. Furthermore, the vast majority of cases were identified in hospital and so are unlikely to be representative of the totality of stroke cases in the community. Only 31 cases from Asiki et al. and 200 from Walker et al. were community based [12, 30]. Further work should include stroke cases identified within the context of management of predominant stroke risk factors such as diabetes and hypertension which can be improved considering the optimal drug regimens available. Interventions targeting accessibility to screening, treatment and sociocultural aspects of health should be considered.

In line with previous research, this review showed that DM is a risk factor for all stroke, but it is more prevalent among those with IS than with HS. Data from previous research has also demonstrated similar findings [8, 34–37]. The INTERSTROKE study was a landmark case-control study, which confirmed that nine modifiable risk factors, including DM, account for approximately 90% of the population's attributable risk for stroke in all regions of the world including SSA [8]. Several other epidemiologic studies [38, 39] have indicated an independent association between DM and IS with a twofold to sixfold increased risk. Diabetes is major risk factor for the development of atherosclerosis and the excess risk of stroke in patients with diabetes is about four times higher when compared with normal individuals in a general population [40]. Poorly controlled diabetes has been shown to contribute to poor outcome in stroke patients [20]. With majority of the diabetic patients living in the developing world, prevention and control of diabetes is becoming a major public health priority [41]. World Health Organization reports [42] have called for more resources to be spent on vascular risk factor reduction in low-income countries and for the development of new ways to provide preventive care.

Results from our study suggest that HIV is a risk factor for all stroke, although it is more prevalent among those with IS rather than HS. A similar finding was observed in a study from Malawi [43]. In Tanzania, the overall prevalence of HIV infection among patients presenting with stroke was 20.9% [44]. It is well recognized that both HIV infection and antiretroviral therapy (ART) could potentially increase an individual's risk of stroke [45]. Given the epidemic of HIV infection and the increasing burden of stroke in SSA, we need large, well designed interventions to control the epidemic, together with integration of care for noncommunicable diseases (NCD) into HIV care programmes.

In the limited data presented here, hypercholesterolemia, smoking, alcohol use, male gender, and hypertension were not significantly associated with stroke. The difference in the pooled prevalences for HS and IS among patients with hypertension, DM, and atrial AF was statistically significant at the 5% level. We did not have any information on the pack years in the studies we analyzed. However, according to previous research, the longer and heavier a person's smoking habit, the higher the risk of stroke [46]. The relationship between the amount of smoking and stroke risk is strongest for ischemic stroke [47]. Although we did not find a significant association between hypertension with all stroke, numerous studies [24, 26] have documented this relationship, and it is consistent throughout SSA. Thus, hypertension is a key risk factor for both HS and IS [9, 48]. Populations in SSA appear to be more at-risk of developing hypertension and subsequent stroke compared to the western world [8, 49]. This difference could be accounted for by a combination of factors, including inadequate funding and lack of infrastructure which often impair diagnosis, screening, treatment and control of hypertension in SSA [9, 50].

## 5. Limitations

Our study has several potential limitations. We could not report the comparative importance of risk factors for HS and IS in SSA because of lack of published data. All included studies in this review were limited to English only from 1980 to 2016, so we may not have included all relevant studies despite our comprehensive search strategy. Other limitations pertain to the quality of the evidence provided by the included studies. Moderate to considerable heterogeneity existed among the studies included in the outcome analysis and all but two of the studies were hospital-based and may not be representative of the totality of stroke in the community. In Tanzania, only a small proportion of stroke cases [12] get to hospital. This highlights the biases associated with hospital-based studies in SSA.

## 6. Conclusions and Recommendations

There remain too few data from SSA to reliably estimate the effect of various factors on the risk of IS and HS. Furthermore, the clear majority of cases were identified in hospital and so are unlikely to be representative of the totality of stroke cases in the community. Further work should include stroke cases identified within the context of management of predominant stroke risk factors such as diabetes and hypertension which can be improved considering the optimal drug regimens available. Interventions targeting accessibility to screening, treatment, and sociocultural aspects of health should be considered.

## Figures and Tables

**Table 1:** Characteristics of case-control studies included in the systematic review.

Author, year	Setting	Study design	Stroke type (IS/HS)	Cases (*n*)	Controls (*n*)	Gender	Hypertension	DM	Alcohol use	Current smoking	HIV sero-positivity	Hypercholesterolemia
Asiki et al. (2015)	Single center- South western Uganda	Case control	Not reported	31	132	+	-	-	-	-	+	-

O'Donnell et al. (2010)	Multicenter- Uganda, Mozambique, South Africa, Sudan, Nigeria	Case control	214/109	323	323	+	+	+	+	+	-	-

Owolabi et al. (2013)	Single center- University College Hospital, Ibadan, Nigeria	Case control	60/30	135	117	+	-	+	+	+	-	-

Benjamin et al. (2015)	Single center- Queen Elizabeth Central Hospital, Blantyre district, Malawi	Case control	Not reported	222	503	+	+	+	+	+	+	+

Walker et al. (2013)	Multicenter - rural Hai district in northern Tanzania, and urban Dar-es-Salaam- Tanzania	Case control	66/14	200	398	+	+	+	+	+	+	+

+ = information available; - = information not available.

**Table 2:** Characteristics of case series included in the systematic review.

Author, year	Country	Patient recruitment and setting	Case definition	Case ascertainment	Stroke type	Mean age (years)	Sample size	IS/HS (%)	Risk factors reported
Mayowa et al., 2013	Nigeria	Hospital based consecutive adult stroke patients	NR	CT scan and/or MRI	Ischemic and hemorrhagic	60.8	282	61.7/33.3	Age, sex, hypertension, waist hip ratio, diabetes, hypercholesterolemia, alcohol use, smoking, physical inactivity, body mass index

Albertino et al., 2010	Mozambique	Hospital based adult stroke admissions	WHO	CT scan and necropsy	Ischemic and hemorrhagic	59.1	651	42.0/36.1	Age, sex, race, hypertension, diabetes, dyslipidaemia, AF

Sennay et al., 2016	Ethiopia	Hospital based adult stroke patients	NR	CT scan and neurological evaluation	Ischemic hemorrhagic, TIA and other	62.8	142	55.6/32.4	Age, sex, hypertension, diabetes, address

Sagui et al., 2005	Senegal	Hospital based adult stroke admissions	WHO	CT scan	Ischemic and hemorrhagic	60.4	107	70.0/30.0	Age, hypertension, diabetes, smoking, hypercholesterolemia, AF

Connor et al., 2007	South Africa	Hospital based consecutive adult stroke patients	WHO	CT scan or MRI	Ischemic and hemorrhagic	53	432	58.0/42.0	Age, male sex, hypertension, diabetes, hypercholesterolemia, alcohol use, smoking, AF, TIA

Nakibuuka et al., 2012	Uganda	Hospital based consecutive adult stroke patients	NR	CT scan	Ischemic and hemorrhagic	62.2	139	77.6/22.4	Age, sex, hypertension, diabetes, smoking, alcohol use, physical inactivity

Masharip et al., 2012	Zambia	Hospital based adult stroke patients	WHO	WHO CT scan	Ischemic and hemorrhagic	55	250	65/35	Age, male sex, hypertension, diabetes, smoking, alcohol use, HIV infection, previous stroke, AF

NR = not reported.

**Table 3:** Risk factors for all stroke expressed as odds ratios with 95% CI values.

Risk factor	*n*	Pooled OR (95% CI)	*I* ^2^ (%)
Hypertension	3	1.66 (0.78-3.55)	88.7
DM	4	**2.39 (1.14–5.03)**	68.2
Alcohol use	4	1.26 (0.68–2.35)	74.2
Smoking	4	1.02 (0.59–1.79)	67.6
HIV infection	3	**2.46 (1.59**–**3.81) **	30.9
Hypercholesterolemia	2	1.18 (0.85–1.63)	0.0

*n* = number of studies.

**Table 4:** Pooled prevalences for hemorrhagic and ischemic stroke by selected risk factors for stroke.

Risk factor	References	Pooled prevalence -HS (% )	Pooled prevalence – IS (% )	*p*
Hypertension	7 [15–17, 24–27]	73.5	62.8	**0.001** ^**∗**^
Diabetes	7 [15–17, 24–27]	10.6	15.9	**0.009** ^**∗**^
Alcohol use	4 [15, 16, 26, 27]	29.3	24.2	0.182
Smoking	6 [15, 16, 24–27]	11.2	13.1	0.340
Atrial fibrillation	5 [15, 16, 24, 25, 27]	2.3	9.6	**0.001** ^**∗**^
HIV infection	2 [15, 16]	11.8	20.0	0.123
Hypercholesterolemia	4 [15, 16, 25, 27]	18.6	13.7	0.176

^*∗*^Statistical significance at 5% level.
